# High temperature tribology of polymer derived ceramic composite coatings

**DOI:** 10.1038/s41598-018-33441-8

**Published:** 2018-10-10

**Authors:** Sajid Ali Alvi, Farid Akhtar

**Affiliations:** Division of Materials Science, Luleå University of Technology, Luleå, 97187 Sweden

## Abstract

Polymer derived ceramic (PDC) composite coatings were deposited on AISI 304 substrates using siloxane based preceramic polymer polymethlysilsquioxane (PMS) and ZrSi_2_ as active filler or Ag as passive filler. The tribological performance of the composite coatings was evaluated at room temperature and moderately high temperatures (150 °C, 200 °C, 300 °C and 400 °C). The composite coatings showed low coefficient of friction (COF), µ, from 0.08 to 0.2 for SiOC-ZrSi_2_ composite coatings, and from 0.02 to 0.3 for SiOC-Ag composite coatings, at room temperature with increasing normal load from 1 to 5 N. High temperature tribology tests showed high COF values from 0.4 to 1 but low wear for SiOC-ZrSi_2_ coating, and low COF from 0.2 to 0.3 for SiOC-Ag coatings at lower temperature ranges. Low load friction tests at room temperature showed negligible wear in SiOC-ZrSi_2_ coatings, suggesting good wear resistant and lubricating properties due to formation of t-ZrO_2_ and carbon. Low COF and high amount of wear was observed in SiOC-Ag composite coatings at room temperature due to high ductility of Ag and smearing of wear debris in the wear track. The coatings and wear tracks were characterized to evaluate the lubrication and wear behavior.

## Introduction

There is an ongoing demand for high temperature tribological coatings in different industries, such as coatings in aerospace (airfoil bearings, various satellite components and rolling element bearing) and automobile (engine bearings, piston assemble and traction drive), with tailored properties, such as high hardness, better friction and wear properties at higher temperatures, as well as oxidation and corrosion resistant properties^1–3^. In order to avoid the cost of designing new high temperature materials, it becomes economically viable to design a coating over conventional materials to sustain material damages at elevated temperatures.

Solid lubricants are utilized for high temperature application because of their low vapor pressure and sublimation. Solid lubricants for high temperature lubrication require good hardness, toughness, oxidation resistant, *in-situ* lubricating phases and high temperature stability^4^. Coatings for lubrication can be either intrinsic, facilitating interfacial shearing of atomic planes, or extrinsic, where additive from surrounding activates the shearing mechanism. However, most of the intrinsic or extrinsic coatings lose their lubricating properties above 300 °C. Therefore, Solid lubricants for higher temperature (>300 °C), such as soft metals (Ag, Cu, Au etc.), fluorides (CaF_2_, BaF_2_), and metal oxides (V_2_O_5_, AgTiO_3_), combined with intrinsic or extrinsic solid lubricants, also known as chameleon coatings, can adapt to different temperature ranges of 25–1000 °C for giving lubricating properties^5^.

Various coating techniques have been explored to enhance the high temperature tribological properties. Laser clad coatings have been developed where coated material forms composite phases with substrate material. Different additives of WS_2_/CaF_2_/h-BN has been added to Ni alloy matrix powders and laser cladded on Ti-6Al-4V substrate tested at different temperatures giving low COF of 0.32–0.35 (25 °C), 0.27–0.3 (300 °C) and 0.21–0.29 (600 °C) and wear rate was reduced to 0.9–9 × 10^−5^ mm^3^/Nm (25 °C), 0.15–8 × 10^−5^ mm^3^/Nm (300 °C) and 2–4 × 10^−5^ mm^3^/Nm (600 °C). The addition of additives formed phase, such as TiC, TiWC_2_, Ti_2_CS and CrS (for WS_2_ addition); TiO_2_, TiC and TiB_2_ (for h-BN addition), resulting in reduced wear rate and improved self-lubricating properties^6–8^. Magnetron sputtering has been explored to obtain thin composite/nanocomposite coatings of oxides and carbides for high temperature tribology. Coated and subsequent *in-situ* formed nanocomposite of yttria-stablized zirconia (YSZ) coating containing Ag and Mo, and binary oxides of α-MoO_3_ and V_2_O_5_ doped with Ag or Cu showed reduced COF from ~0.7 to 0.14 due to formation of magneli phases with increasing temperature up to 700 °C^9–14^. Ternary oxides of (Ag, Cu)-(Ta, W, Mo)-O have also been developed with magnetron sputtering to obtain good lubricating and wear properties at high temperatures, such as AgTaO_3_ (750 °C; COF: 0.06; wear rate: 4 × 10^−7^ mm^3^/Nm), CuTa_2_O_6_ (750 °C; COF: 0.24; wear rate: 0.7 × 10^−7^ mm^3^/Nm), Ag_2_Mo_2_O_7_ (600 °C; COF: 0.12), and Ag_2_WO_4_ (600 °C; COF: 0.43)^15–17^. Atomic layer deposition, with its ability to grow controlled coating layers, has been explored for high temperature tribology to develop nanocrystalline ZnO (room temperature(RT); COF: ~0.1), wear resistant nanolaminates of ZnO/Al_2_O_3_/ZrO_2_ with a thickness of 140 nm (wear rate at 150 °C: 3.6 × 10^−6^ mm^3^/Nm; 400 °C: 3.1 × 10^−5^ mm^3^/Nm)^18–20^. Deposition methods, such as physical vapor deposition (PVD) and laser deposition, have been used to develop lubricious ZnO coatings (RT; COF: 0.1–0.2), wear resistant composite coatings of NiCrAlTi/TiC/Ti-CaF_2_ (at 300 °C, wear rate: 5 × 10^−5^ mm^3^/Nm; and at 600 °C, wear rate: 2 × 10^−5^ mm^3^/Nm) and duplex treated nitride/TiAlN coatings (wear rate at 400 °C: 7 × 10^−6^ mm^3^/Nm, at 500 °C: 4 × 10^−6^ mm^3^/Nm, and at 600 °C: 8 × 10^−6^ mm^3^/Nm)^21–23^.

Ceramic coatings obtained from silicon-based polymer derived ceramics (PDCs) offer a simple, energy efficient robust technique to obtain coatings with tailored properties for larger and complex substrates. PDC coatings are obtained using organo-silicon precursors, such as polysiloxane^24–27^, polysilazane^28–32^, polycarbonsilane^33–35^ etc., to obtain SiOC, SiCN/Si_3_N_4_/SiOCN/SiC, and SiC coatings, respectively, after crosslinking and pyrolysis. The polymer-to-ceramic conversion is obtained at lower temperature of up to 800 °C, making it economical and environmentally effective compare to other coating techniques. The advantages of PDC coatings have been identified due to its inexpensive deposition techniques (such as dip coating, spin coating or spray coating), easier deposition with starting liquid phase, control over properties through nanostructure phases, and the ability to coat complex shapes^36^. The main drawback arises with PDC coating deposition from shrinkage during polymer to ceramic conversion, restricting coating thickness of pure PDC to only few microns. This problem has been overcome by using active or passive filler to compensate for the shrinkage arising from evaporation of volatile hydrocarbon and other compounds. Passive filler, such as BN, ZrO_2_, TiO_2_ and SiC, can reduce the volume fraction of shrinkage phase, whereas active fillers, such Ti, Al, ZrSi_2_, TiSi_2_ and TiB_2_, can react with gaseous products or atmosphere to form new phases^37^. Fillers may also modify the properties of coatings, such as thermal and electrical conductivity (such as graphite or ZrO_2_), thermal expansion (such as ZrO_2_), hardness (such as SiC and c-BN), and tribology (such as h-BN and c-BN). Recently, there have been few studies performed at room temperature and very low loads, in the range of mN, for lubricating behavior of coated/bulk PDCs^38–42^, and on composite PDC coatings with addition of h-BN and c-BN fillers^43^. However, there are no reports studying the tribological properties of PDC coatings at higher loads and temperatures to best of our knowledge. In this regards, the current work focuses on the design of PDC composite coatings that offer superior tribological properties at higher loads and temperatures. PDC composite coatings were designed using fillers, such as ZrSi_2_ and Ag, which can give tribological properties at different temperatures.

## Results and Discussion

**Figure 1:**
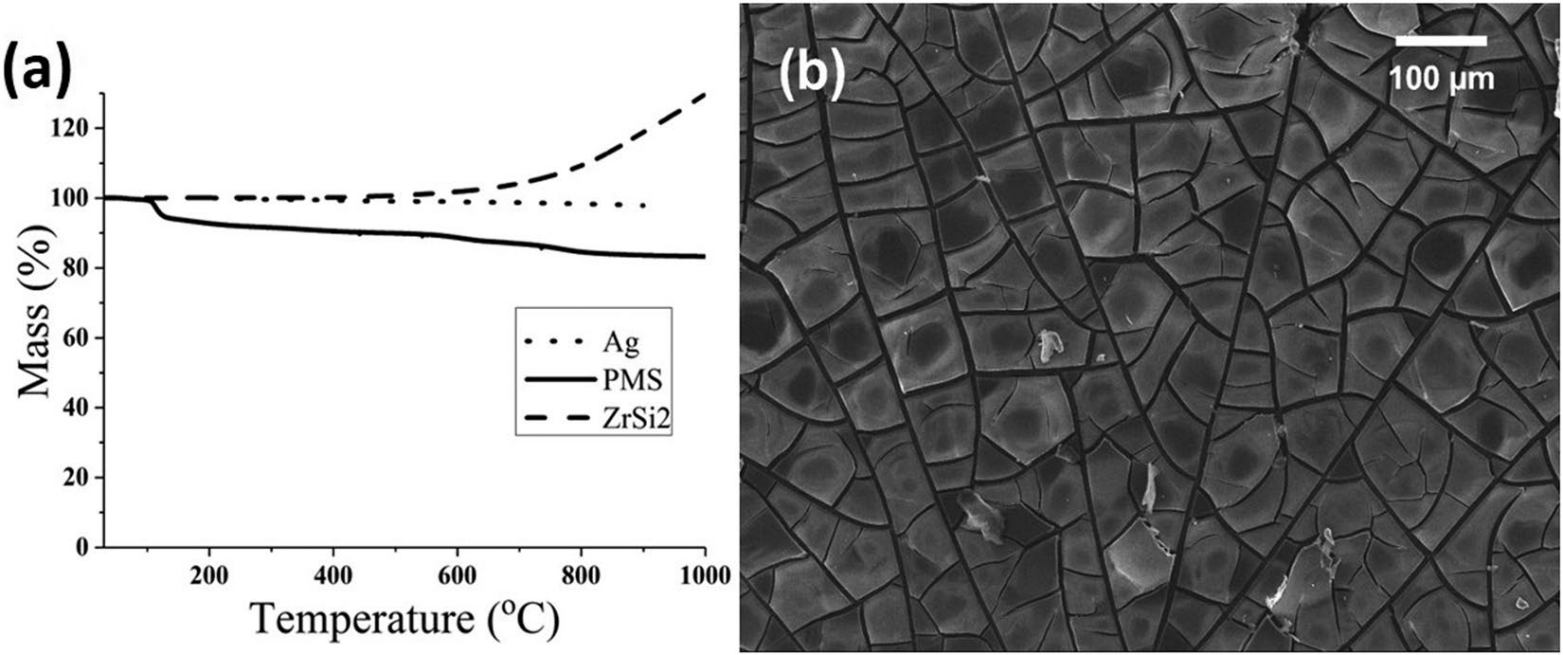
(**a**) TGA of PMS polymer precursor, Ag and ZrSi_2_ powder, (**b**) PMS coating without filler after pyrolysis.

Thermal gravimetric analysis (TGA) of PMS polymer precursor in Fig. 1a shows three steps of ceramic conversion process giving ceramic yield of 83 wt.% on complete conversion to SiOC ceramic at 800 °C. According to previous reports on PMS conversion to ceramic, in the first step (110–370 °C) cross-linking occurs with the release of isopropanol and H_2_O; in the second step (460 to 670 °C) oligomers as wells as ethanol evolve; and in the third step (650–1000 °C) conversion to SiOC completes with the release of H_2_, methane and trace amount of H_2_O^44^. Thermal behavior of ZrSi_2_ showed a mass gain of 30 wt.% till 1000 °C. According to Gebwein *et al*.^45^, the mass gain of ZrSi_2_ is related to oxidation, which starts at 500 °C and completes at 1000 °C with the formation of SiO_2_ and ZrO_2_ phases. They have suggested that ZrSi_2_ oxidizes partially according to Wagner theory of selective oxidation^46^, where less nobel element (Zr) is oxidized preferentially along with diffusion of Si atoms to bulk of material forming elemental silicon. In addition to this, non-preferential oxidation takes place up to 900 °C as shown in the reaction mechanism below:

1 $${{\rm{ZrSi}}}_{2}\to {{\rm{ZrO}}}_{2}+{{\rm{SiO}}}_{2}+{\rm{Si}}\to {{\rm{ZrO}}}_{2}+{{\rm{SiO}}}_{2}$$

Thermal behavior of Ag showed weigh loss of 2 wt.% over the temperature range till 800 °C due to desorption of water. The mass loss of PMS during ceramic conversion process results in formation of cracks and delamination of coatings, as shown in Fig. 1b. This happens due to the severe increase in density and volume shrinkage on conversion to ceramic, thus limiting the PDC coating thickness without the aid of fillers^47^. In order to compensate for mass loss, 5 vol.% ZrSi_2_ and Ag was added as a filler material to PMS to prepare films with less defects after pyrolysis.

**Figure 2:**
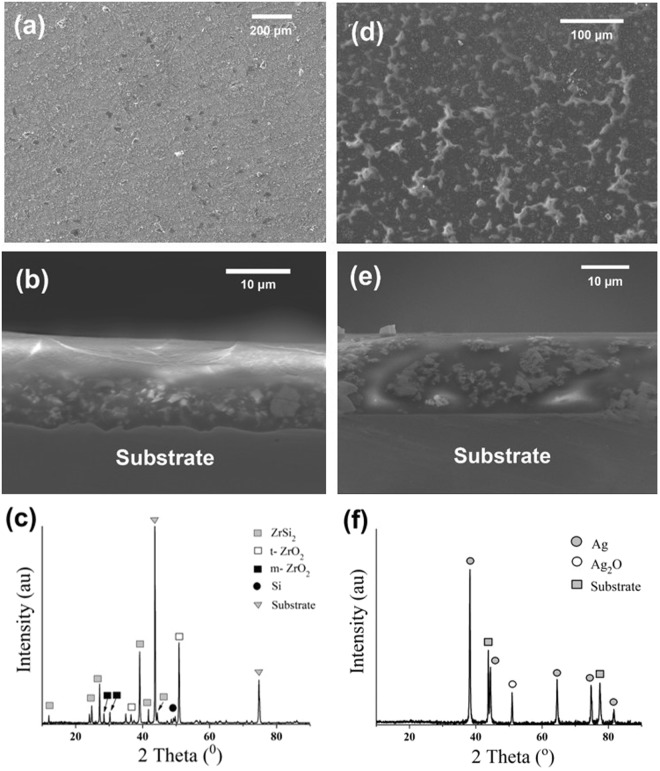
Characterization of SiOC-ZrSi_2_ (**a**–**c**) and SiOC-Ag (**d**–**f**) coatings pyrolyzed at 700 °C. (**a**,**d**) SEM images of top surface, (**b**,**e**) SEM images of cross-section, (**c**,**f**) X-ray diffraction analysis.

Composite coatings (pyrolyzed at 800 °C, Ar atmosphere) showed relatively high amount of cracks and therefore were not analyzed further, see Supplement Information S1. The coatings pyrolyzed at 700 °C, with similar experimental conditions, showed crack-free microstructure and partial oxidation of fillers to form t-ZrO_2_ and Ag_2_O phases, as seen in Fig. 2. The residual content of incomplete polymer was calculated to be around 5 wt% from TGA The top view of the SiOC-ZrSi_2_ coating (Fig. 2a) shows a uniform crack-free coating after pyrolysis. However, SiOC-Ag coating (Fig. 2d) shows presence of aggregates on the surface. Such aggregates form in SiOC-Ag coatings due to its addition to polymeric solution with high viscosity. The aggregation of Ag nanoparticles has been attributed to the attractive forces created by van der Waals forces or chemical bonds between nanoparticles^48^. Whilst, ZrSi_2_ act as an active filler and counters the formation of cracks and aggregation in SiOC-ZrSi_2_ coatings^49,50^. The cross-section of the composite coatings revealed a uniform coating thickness of ~12 µm, Fig. 2(b,e), where ZrSi_2_ is distributed uniformly and Ag is aggregated, which tends to happen during its mixing with polymeric precursor. Microhardnes of SiOC-ZrSi_2_ coating was found to be 200 Hv, as shown in Fig. S11. The optical profilometry of SiOC-ZrSi_2_ composite coating showed that the coating surface had a surface roughness of 800 nm, as shown in Supplementary Information S2. The X-ray diffraction (XRD) data of SiOC-ZrSi_2_ coating show phases of ZrSi_2_, SiO_2_ and tetragonal-ZrO_2_ (t-ZrO_2_) (Fig. 2c); while of SiOC-Ag coatings show presence of Ag and Ag_2_O (Fig. 2f).

The oxidation of Ag to Ag_2_O has been studied by X. Bao *et al*.^51^ at higher temperatures, showing the formation of Ag_2_O using x-ray photoelectron spectroscopy (XPS), where the binding energy and O/Ag ratio changes from 530 eV and 0.2 at 300 °C to 529 eV and 0.5 at 600 °C, respectively, which corresponds to formation of stoichiometric Ag_2_O. The oxidation reaction of Ag nanoparticles takes place by:

2 $$2{\rm{A}}{\rm{g}}+\frac{1}{2}{{\rm{O}}}_{2}\to {{\rm{A}}{\rm{g}}}_{2}{\rm{O}}$$

Although, the complete ceramization of PMS preceramic polymer takes place at 800 °C, the coating was pyrolyzed at 700 °C to combine the properties of ceramic coating with incomplete precursor conversion to enhance lubricating properties. Formation of sp^2^ carbon has been reported during the polymer-to-ceramic conversion of PDC, which can be particularly advantageous to the lubricating properties^52^. Furthermore, t-ZrO_2_ and SiO_2_ phases observed arise from the partial oxidation of ZrSi_2_, which has been reported to be beneficial towards load bearing capability^53^.

The energy dispersive x-ray spectroscopy (EDS) mapping of SiOC-ZrSi_2_ and SiOC-Ag coating cross-section is shown in supplementary information, Fig. S3. High amount of elemental zirconium can be observed at lower section of coating as ZrSi_2_ with 2–3 µm particle sizes are placed near the substrate, and top coating mostly consists of Si, O and C. The formation of Ag aggregates in SiOC-Ag coating is evident in EDS mapping.

**Figure 3:**
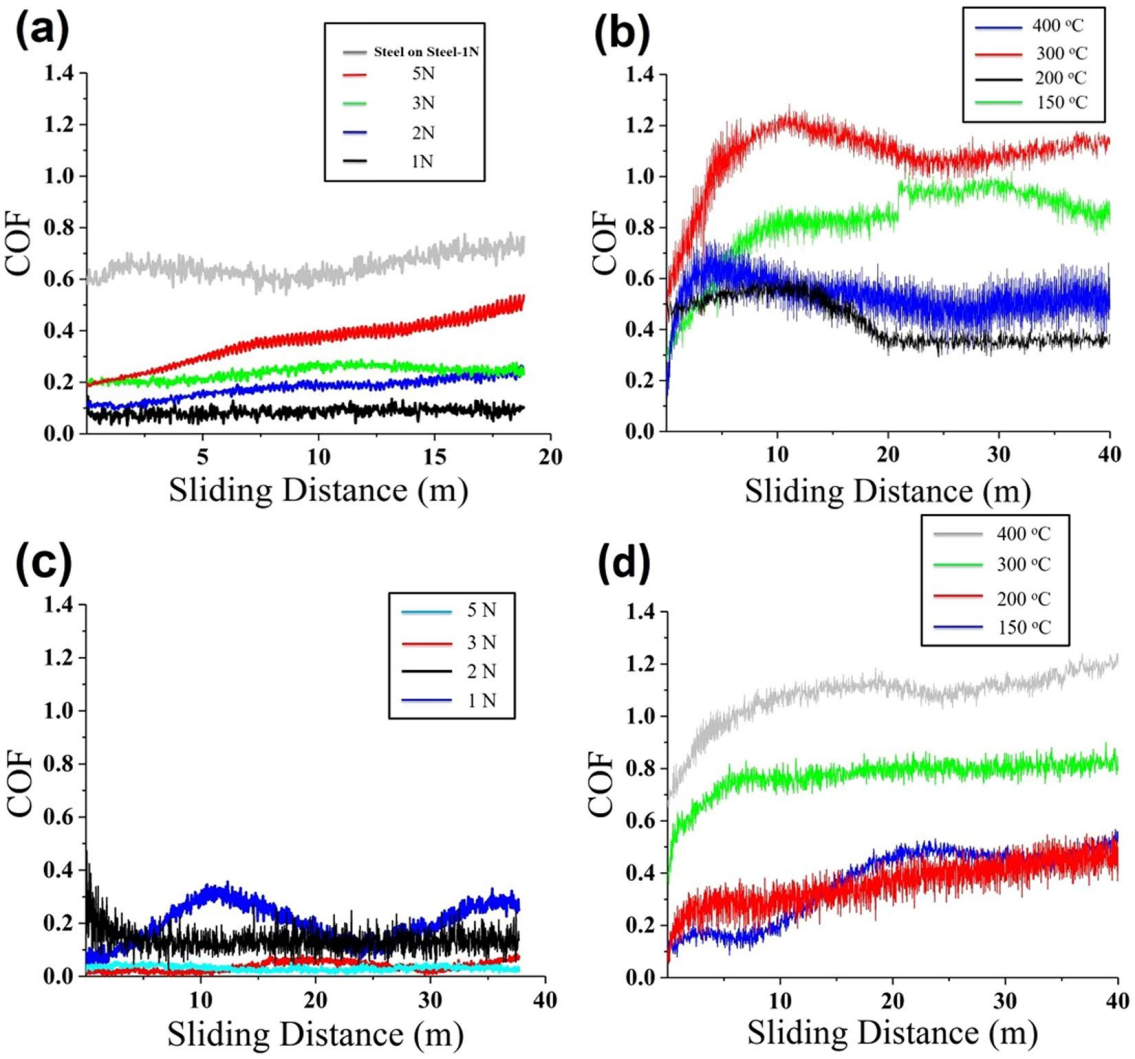
Friction coefficient curves of SiOC-ZrSi_2_ and SiOC-Ag composite coating: (**a**) SiOC-ZrSi_2_ at room temperature with various loads, and (**b**) SiOC-ZrSi_2_ at higher temperatures with 1 N load; (**c**) SiOC-Ag at room temperature with various loads, and (**d**) SiOC-Ag at higher temperatures with 1 N load.

The friction coefficient versus the sliding distance curves of SiOC-ZrSi_2_ and SiOC-Ag coatings with varying loads and at various temperatures are shown in Fig. 3. Steel ball on steel substrate friction test at 1 N normal load showed an average COF of 0.66, as shown in Fig. 3a. The wear track of steel-on-steel showed high amount of abrasive and adhesive wear along with high amount of transfer layer on steel counter ball, as shown in Fig. S9a,b, respectively. SiOC-ZrSi_2_ composite coating, at room temperature with 1 N load, shows the lowest COF of 0.08, which increases with increasing load from 1 N to 5 N from 0.08 to 0.5, as shown in Fig. 3a. It has been reported in the literature that the polymer derived SiOC containing high amount of carbon with sp^2^ structure can act as self-lubricant layer by formation of a thin transfer layer on the ball surface^39,54^. Furthermore, the presence of t-ZrO_2_ and SiO_2_ (Fig. 2e) may enhance the load bearing properties^45,53^. The friction test performed at a higher load of 5 N shows that the coating was intact after 20 m sliding distance. The COF increased to 0.5, however it was lower than the steel-to-steel friction coefficient of 0.66. The low COF and stability of the coating is attributed to the formation of sp^2^ carbon, as determined by XRD after polymer to ceramic conversion^52^ and elemental Si (Fig. 2c) and incomplete (5 wt% retained polymer, as determined by TGA) conversion of polymeric precursor.

**Figure 4:**
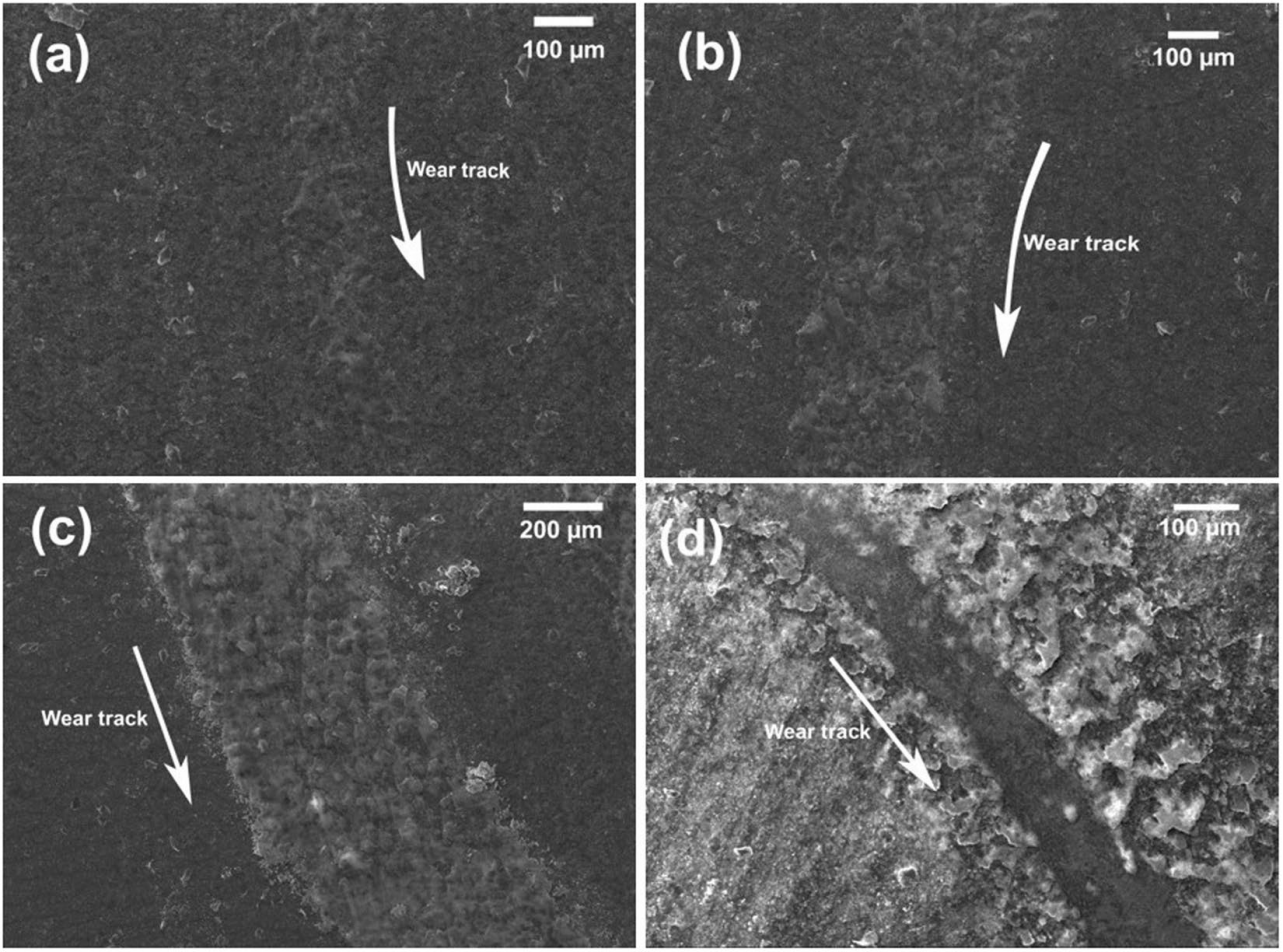
SEM image of wear tracks from SiOC-ZrSi_2_ at room temperature with a sliding distance of 18 m at (**a**) 1 N load; (**b**) 2 N load; (**c**) 3 N load; and (**d**) 5 N load.

**Figure 5:**
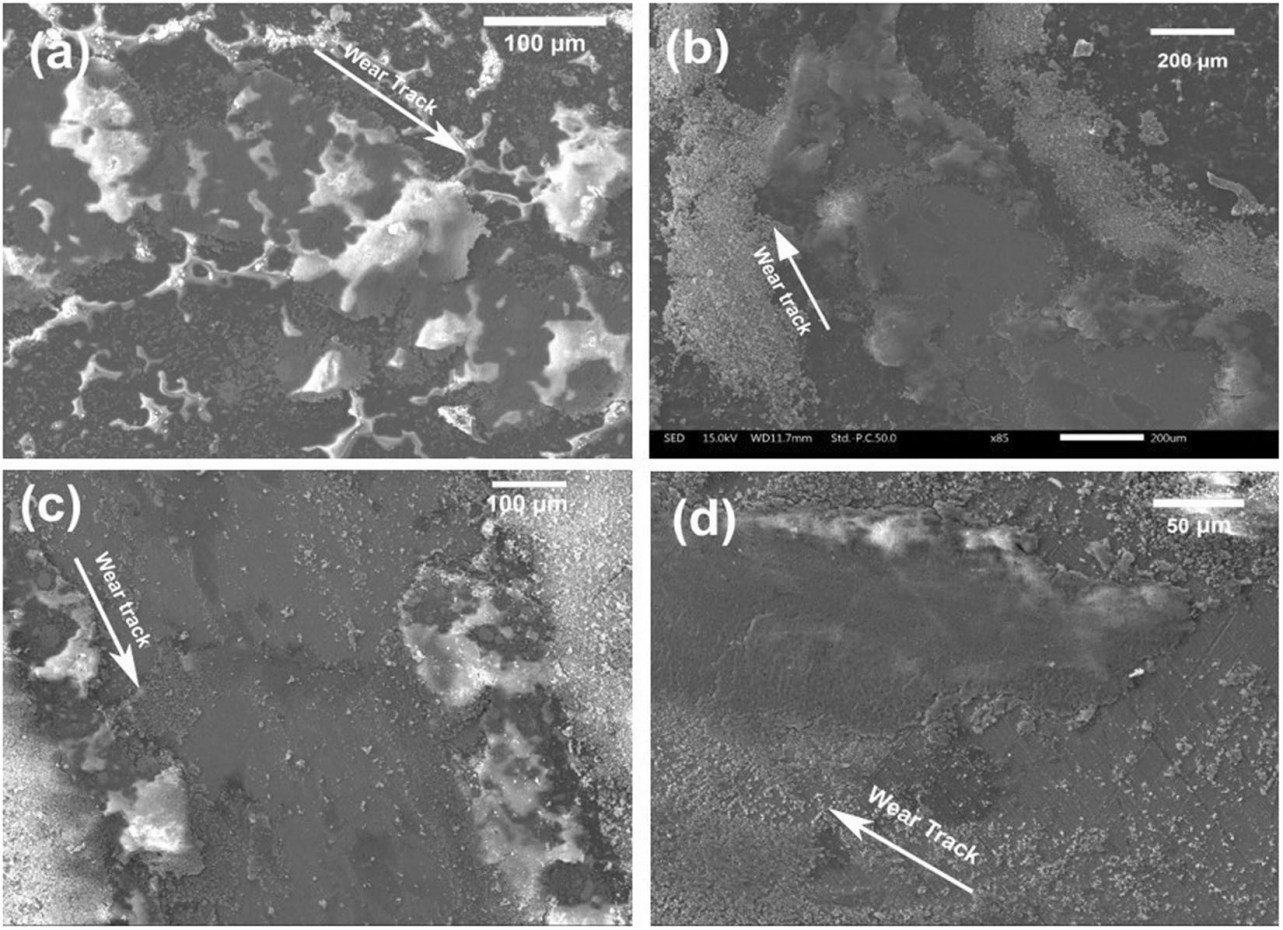
SEM micrographs of wear tracks of SiOC-Ag composite coating: (**a**) 1 N load; (**b**) 2 N load, (**c**) 3 N load; and (**d**) 5 N load.

The morphology of wear tracks and counter steel ball after friction tests on SiOC-ZrSi_2_ coatings at room temperature at various loads can be seen in Fig. 4(a–d) and in supplementary information Fig. S4, respectively. Wear tracks after friction tests at 1 N, 2 N and 3 N load (Fig. 4a–c) and corresponding EDS (Fig. S5) showed that there was less wear of the coating with low amount of iron (Fe) present in the wear track. On increasing load to 5 N, extensive wear of the coating is visible (Fig. 4d). The corresponding EDS (Fig. 5S, site 4) confirms the presence of coating after the test. The scratch test showed an adhesion of 6 N, as shown in Fig. S10, after which an abrupt increase in COF was caused by coating failure. This suggests good adhesion of coating to the substrate even with wear at higher load. The strong adhesion is attributed to the direct chemical bonding between the substrate and polymeric precursor as PMS reacts with hydroxyl groups present at the steel surface, forming metal-O-Si bonds^55^. The microstructure and EDS analysis of steel counter balls show negligible coating transfer after friction tests at lower load, as shown in S4.

Friction curves (Fig. 3c) and corresponding wear tracks (Fig. 5a–d) summarize the performance of SiOC-Ag composite coatings at room temperature and various loads. The friction test at 1 N showed a varying COF between 0.08 and 0.3, which could be related to the slipping and gripping of counter ball with Ag and SiOC. The average COF decreased to 0.15, 0.04 and 0.02 at loads of 2 N, 3 N and 5 N, respectively. The very low COF values are related to smearing effect of Ag in the coating and lubrication through transfer of coating to counter balls. Lubrication through coating transfer is observed on the counter ball, where the amount of transferred coating increased with increasing loads, as shown in supplementary information Fig. S8 and corresponding EDS analysis in Table S8. The wear track resulting from 1 N load, Fig. 5a, shows low amount of wear; whereas, coating removal is observed at higher loads in addition to smearing of Ag in the wear tracks, as shown in Fig. 5b–d. In contrast to friction properties of SiOC-ZrSi_2_ coatings, SiOC-Ag has lower load bearing capability.

**Figure 6:**
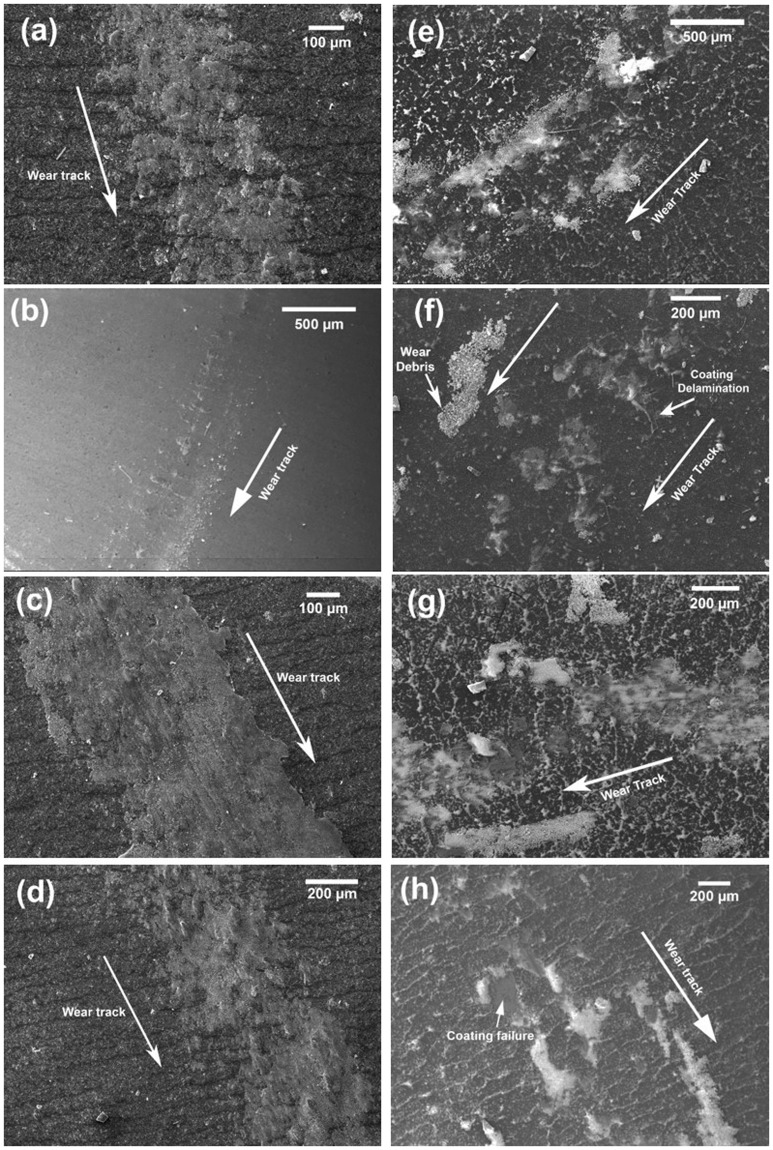
SEM images of wear tracks of SiOC-ZrSi_2_ composite coatings with 1 N load at different temperatures for: (**a**) 150 °C, (**b**) 200 °C, and (**c**) 300 °C, (**d**) 400 °C; and SiOC-Ag composite coatings: (**e**) 150 °C; (**f**) 200 °C; (**g**) 300 °C, (**h**) 400 °C.

High temperature friction tests and corresponding wear tracks of SiOC-ZrSi_2_ coatings are shown in Fig. 3b and Fig. 6(a–d), respectively. It can be seen that friction tests at 150 °C and 300 °C show higher COF values of 0.9 and 1.1, respectively, which are higher than COF of steel of 0.6–0.8. On increasing the temperature to 200 °C and 400 °C, the COF reduces to 0.4 and 0.45 after initial high COF due to run-in period, as shown in Fig. 3b. The subsequent reduction in friction can be related to transferred coating lubrication, which consequently reduces the friction between counter steel ball and coated substrate^56^. The wear tracks of the corresponding high temperature friction test showed low wear where coating was not completely removed, as shown in Fig. 6(a–d). It can be observed from EDS analysis of the wear track, as shown in Fig. S6, that the coating was sheared over the wear track during all friction tests, which can be related to the softening of unconverted polymeric phase in the coatings at higher temperature. The EDS analyses of the wear tracks show the presence of high amount of Si, O, and Zr and small amount of Fe over wear tracks (S6), suggesting that coating wasn’t removed during the test. The coating transfer layers analyzed with EDS analysis on the counter steel ball after high temperature friction tests showed that the amount of transfer layers increased with increasing test temperatures and subsequently shielding the ball surface from contact with specimen surface to give low friction and wear, as shown in S7. The highest amount of transferred coating material was observed during 400 °C friction test, which resulted in reduced COF compare to other temperature tests.

Similarly, high temperature friction tests were performed at SiOC-Ag composite coatings at temperatures of 150 °C, 200 °C, 300 °C, and 400 °C, as shown in Fig. 3d. In contrast to high temperature friction tests over SiOC-ZrSi_2_ coatings, SiOC-Ag composite coatings showed low average COF in the range of 0.2 to 0.5 at lower temperature of 150 °C and 200 °C, and coating transfer was observed after 1–2 m of initial sliding. The wear tracks corresponding to high temperature friction tests showed high wear, delamination of coatings at different places along with smearing of Ag in the wear track as well as in the delaminated coating regions, as shown in Fig. 6(e–h).

The high temperature tribology tests of PDC composite coatings suggest that the use of active filler ZrSi_2_ enhances the load bearing and lubricating properties of coatings up to 4 N normal loads. Whereas, addition of passive filler, Ag nanoparticles, enhances the lubricating properties through transfer coating lubrication at room temperature as well as high temperature friction but limits the applied load to 1 N.

In conclusion, two types of ceramic filler/PDC composite coating were developed in this work using spin coating to enhance the tribological properties at room temperature and to some extend at high temperature (400 °C). PDC composite coatings of SiOC-ZrSi_2_ and SiOC-Ag with a thickness of ~12 µm was obtained using spin coating. SiOC-ZrSi_2_ showed low COF from 0.08 to 0.3 with increasing load from 1 to 5 N load. High temperature friction test on SiOC-ZrSi_2_ showed a decreasing trend in COF with increasing temperature to 400 °C. Such high load bearing in SiOC-ZrSi_2_ has been suggested due to the formation of t-ZrO_2_ from partial oxidation of active-filler ZrSi_2_, formation of sp^2^ carbon network and partial conversion of polymer to ceramic. The friction tests on SiOC-Ag coatings revealed decreasing average COF values from 0.2 to 0.02 with increasing loads at room temperature from 1 to 5 N. The low COF in SiOC-Ag composite coatings arises due to Ag lubricating properties and smearing effect of Ag wear debris in the wear track. The high temperature friction tests of SiOC-Ag composite coating showed low COF in the range of 150–200 °C over constant low load due to low shear strength of Ag. However, the tribological property of Ag becomes poor due to excessive softening at 300 °C.

## Methods

### Coating Deposition

Coatings of SiOC-ZrSi_2_ and SiOC-Ag investigated in this work were developed using commercially available polymeric precursor polymethylsilsquioxane (PMS, MK Belsil, PMS, Wacker, GmbH, Germany), zirconium disilicide (3 µm, US Research Nanomaterials Inc., USA) as active filler material, and silver (70–80 nm, US Research Nanomaterial Inc., USA) as passive filler material. Polymeric suspension was developed using PMS (23 vol%), isoproponal (72 vol%, IPA), ZrSi_2_ and Ag (5 vol%). PMS precursor was first dissolved in IPA with magnetic stirrer, followed by addition of Ag or ZrSi_2_ and further mixing. The resulting suspensions were ball-milled for 1 hour (ZrSi_2_ filler) and 24 hours (Ag filler) to homogenize the mixture. AISI 304 stainless steel sheets was used as a substrate material. The substrate sheets were polishing with SiC papers to achieve surface roughness of 1 µm for better adhesion, followed by cleaning with ethanol in sonicator and drying in oven at 120 °C for 2 hour. Polymeric suspension was deposited on steel substrate using spin coater at 1000 rpm for 10 seconds. The resulting coatings were cross-linked at 190 °C for 1 hr in air using Nabertherm box furnace, followed by pyrolysis at 700 °C for 1 hr in argon atmosphere using Nabertherm tube furnace to convert PMS preceramic polymer to SiOC ceramic.

### Coating Characterization

The coating microstructure and elemental composition were characterized using scanning electron microscopy (SEM, JSM-IT300LV, JEOL GmbH, Germany) and energy dispersive x-ray spectroscopy (EDS) using working distance of 10 mm and accelerating voltage of 15 kV. For EDS analysis of wear tracks, low accelerating voltage of 10 kV was used to lower the interaction volume on the surface and area analysis of around 200 × 200 µm was used. EDS area analysis was performed at 6 different places around each wear track. Thermal behavior of pre-ceramic polymer, ZrSi_2_, and Ag was analyzed till 1000 °C with a heating rate of 10 °C/min in air atmosphere using thermogravimetric analyzer (TGA 8000, Perkin Elmer, USA). The phases in coating were identified using Cu Kα radiation X-ray diffraction with monochromator (PANalytical Empyrean). Microhardness of coating was measured using Vickers microhardness. The surface of coating was analyzed using optical profiler (Wyko 1100 3D) by considering the Ra parameter of the coating.

### Tribological Characterization

Friction tests were performed in air atmosphere with relative humidity of 50% using steel ball-on-disc modular-tribometer (Rtec Universal Tribometer, San Jose, USA) at room temperature (22 °C) and high temperatures (150 °C, 200 °C, 300 °C, and 400 °C). AISI 304 stainless steel substrate with a dimension of 15 mm × 15 mm was used for room temperature tests and a circular disc with 50.8 mm diameter for high temperature tests. Counter ball of E52100 alloy steel (Grade 25) with a hardness 60–66 HRC; and diameter of 6.5 and 9.5 mm for room temperature and high temperature friction tests, respectively, were used. The normal load was varied from 1 to 5 N, corresponding to maximum hertzian contact pressure of 448 to 767 MPa for steel ball on steel substrate, and a constant speed of 100 rpm and 50 rpm was applied for 10 minutes timespan for room temperature and high temperature tests, respectively. The friction tests were repeated with at least 4 coatings of each composition to obtain repeatability and their statistics are stated in Table S12. These experimental conditions have been chosen for applications, such as MEMS, where low load (mN) is applied.

## Electronic supplementary material

Supplementary Information
