# Supplementary material for: High temperature tribology of polymer derived ceramic composite coatings
Source: Sci Rep. 2018 Oct 10;8:15105. doi: 10.1038/s41598-018-33441-8 (PMC6180137; doi:10.1038/s41598-018-33441-8)
Supplement: Supplementary file 1 — Supplementary Information [file 41598_2018_33441_MOESM1_ESM.docx]

**Supplementary Information**

**High temperature tribology of polymer derived ceramics composite coatings**

1. Sajid Ali Alvi^1, a^**,** and Farid Akhtar^1, b *^
2. ^1^Division of Materials Science, Luleå University of Technology, Luleå, Sweden
3. ^a^sajid.alvi@ltu.se, ^b^farid.akhtar@ltu.se
4. * Corresponding author

**S1:**

| 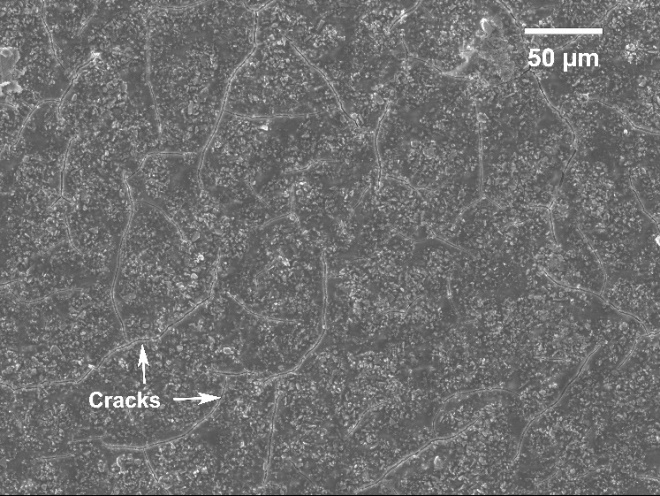 |
| --- |
| Figure S1: Top surface of SiOC-ZrSi_2_ coating pyrolyzed at 800 ^o^C. |

**S2:**

| 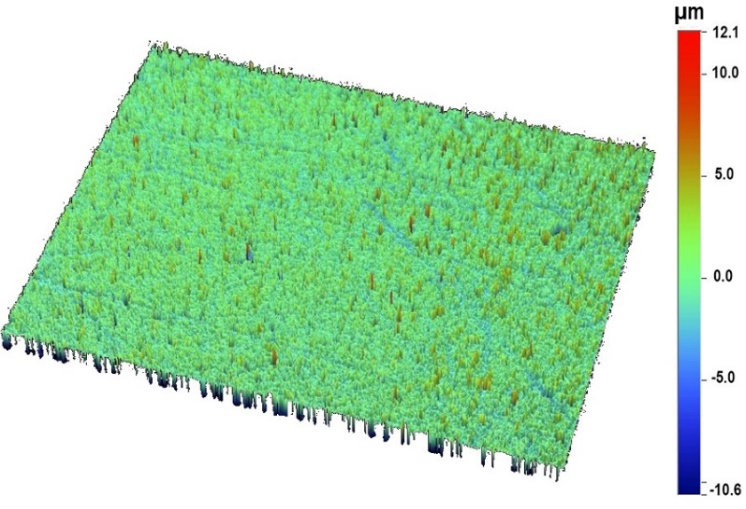 |
| --- |
| Figure S2: Optical profilometry of SiOC-ZrSi_2_ coating pyrolyzed at 700 ^o^C |

**S3:**

| 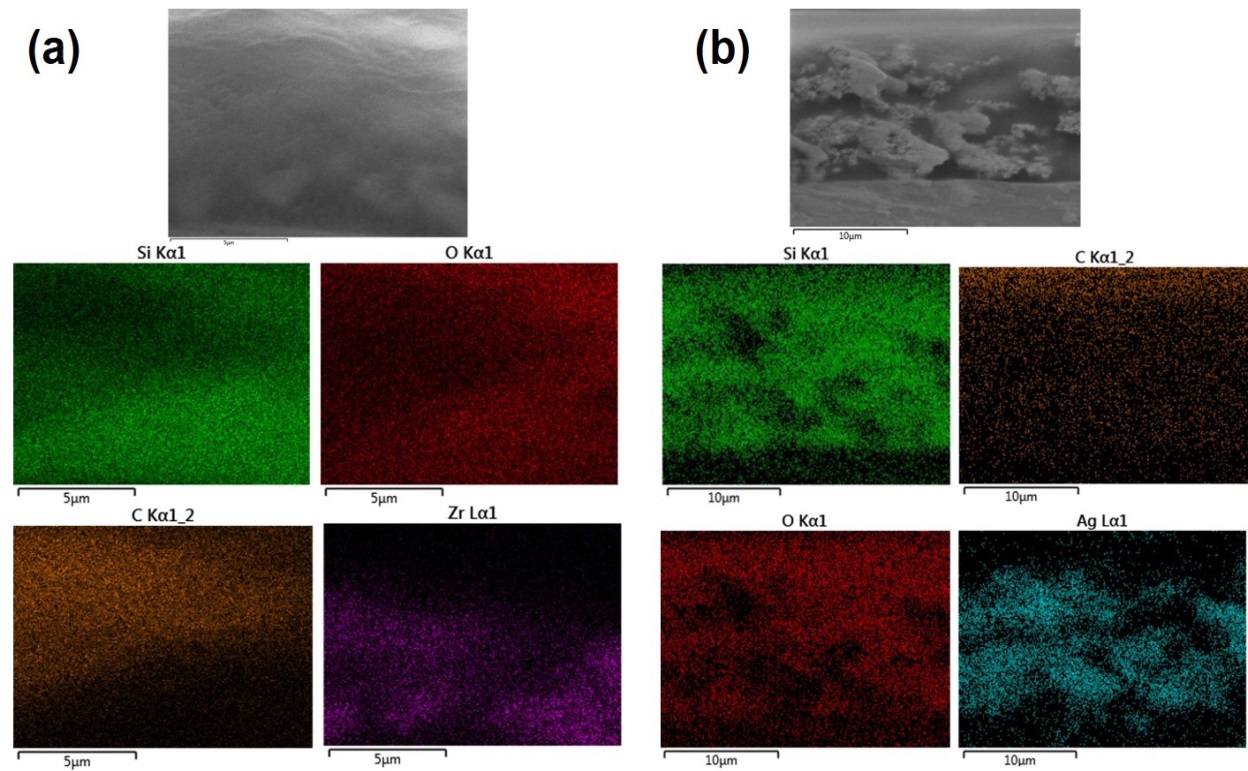 |
| --- |
| Figure S3: Elemental mapping of coating cross-section (a) SiOC-ZrSi_2_ coating, and (b) SiOC-Ag coating |

Counter ball from 1 N and 2 N friction tests showed some amount of wear with no coating transfer, which was confirmed by EDS analysis in S4 (site 1 & 2), suggesting good stability of the coatings. At higher loads of 3 N and 5 N, the amount of transferred coating is increased (S4- site 3 & 4), suggesting decrease in stability of coatings.

**S4:**

| **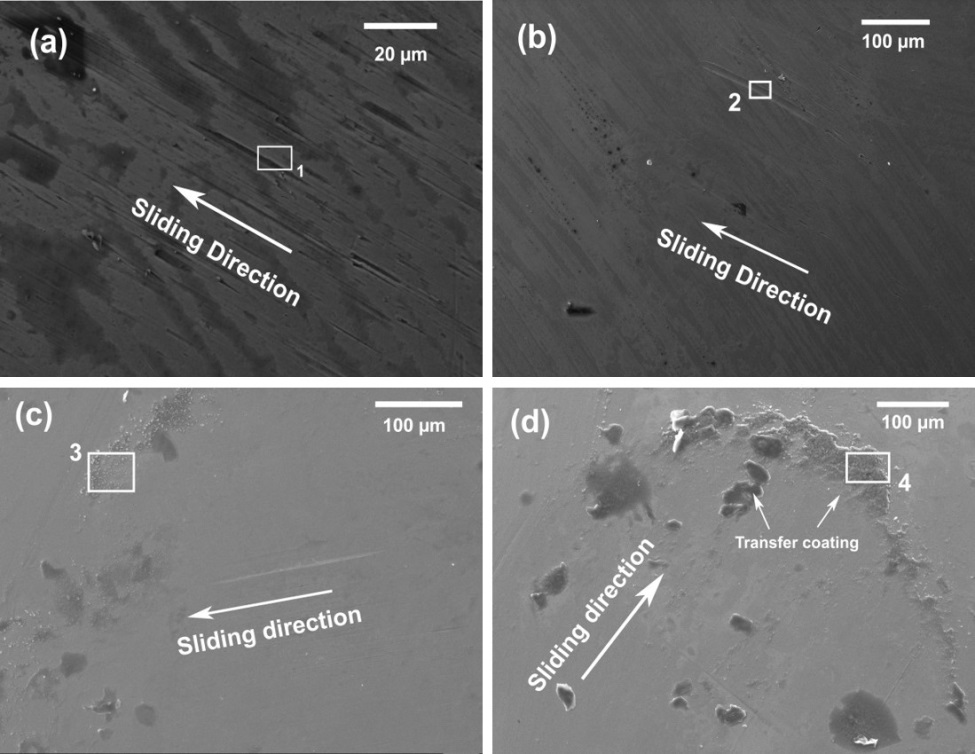** |  |
| --- | --- |
| Figure S4: SEM images of counter ball from SiOC-ZrSi_2_ composite coating after friction tests: (a) 1N load; (b) 2 N load; (c) 3 N load; and (d) 5 N load. |  |
| 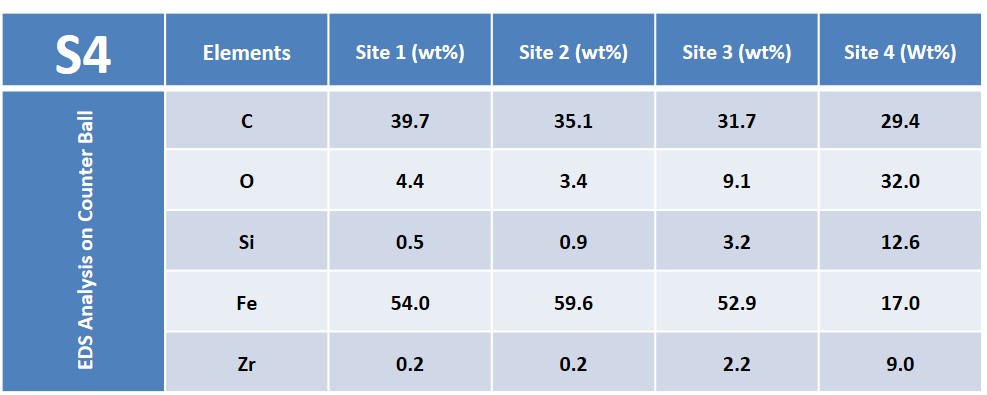 | |
| Table S4: EDS of SEM images of counter ball in Figure S5 from SiOC-ZrSi_2_ composite coating after friction test at room temperature: (site 1) 1N load; (site 2) 2 N load; (site 3) 3 N load; and (site 4) 5 N load. | |

**S5:**

| **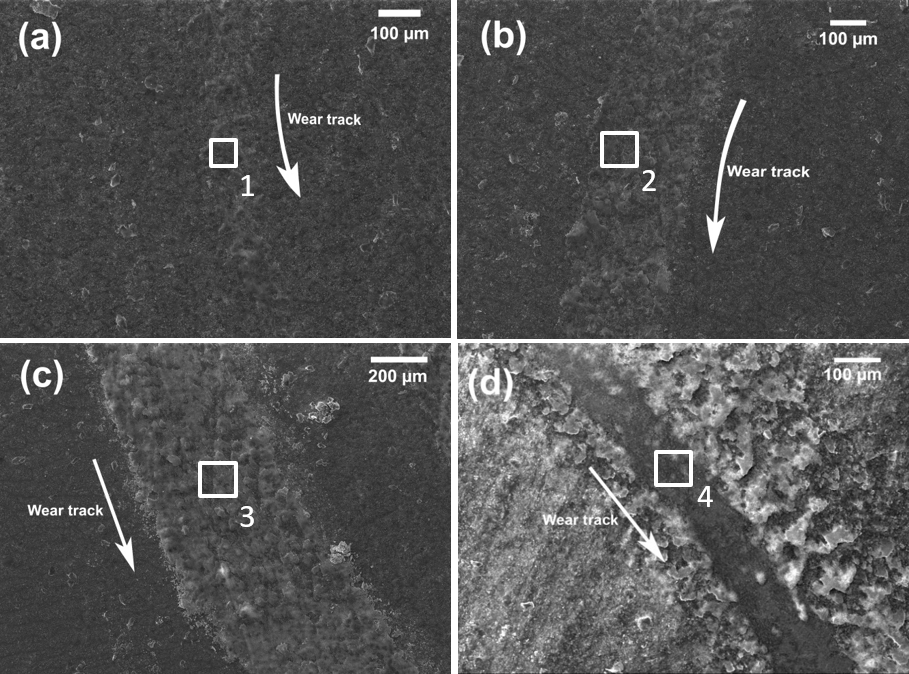** |
| --- |
| Figure S5: EDS sites on wear track of SiOC-ZrSi2 coating at room temperature a) 1N b) 2N c) 3N d) 5N. |

| **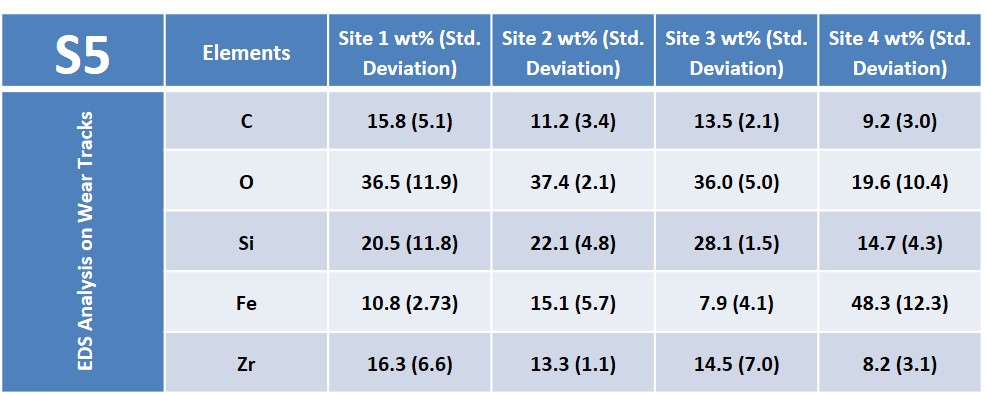** |
| --- |
| Table S5: EDS and standard deviation in wear tracks in Figure S5 from SiOC-ZrSi_2_ composite coating after friction tests at: (site 1) 1N; (site 2) 2N; (site 3) 3N; (site 4) 5N. |

**S6:**

| **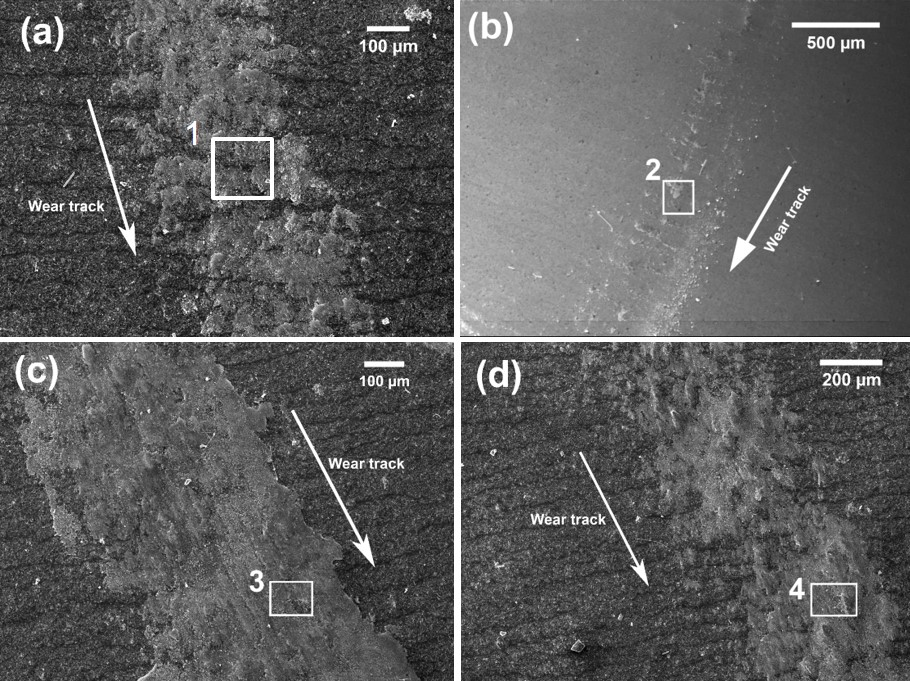** |
| --- |
| Figure S6: wear tracks from SiOC-ZrSi_2_ composite coating after friction tests at 1N load at different temperatures (a) 150 ^o^C; (b) 200 ^o^C; (c) 300 ^o^C; and (d) 400 ^o^C. |

| **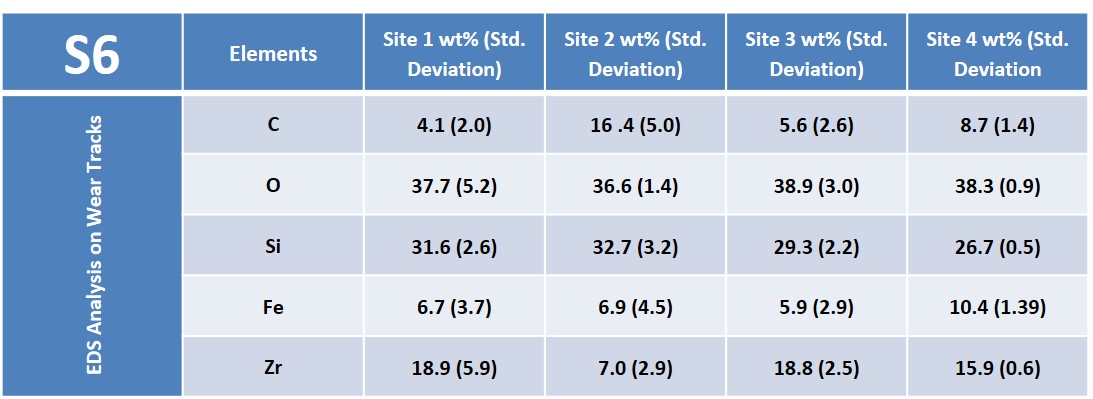** |
| --- |
| Table S6: EDS and standard deviation in wear tracks in Figure S6 from SiOC-ZrSi_2_ composite coating after friction tests at 1N load at different temperatures (Site1) 150 ^o^C; (site 2) 200 ^o^C; (site 3) 300 ^o^C; and (site 4) 400 ^o^C. |

**S7:**

| **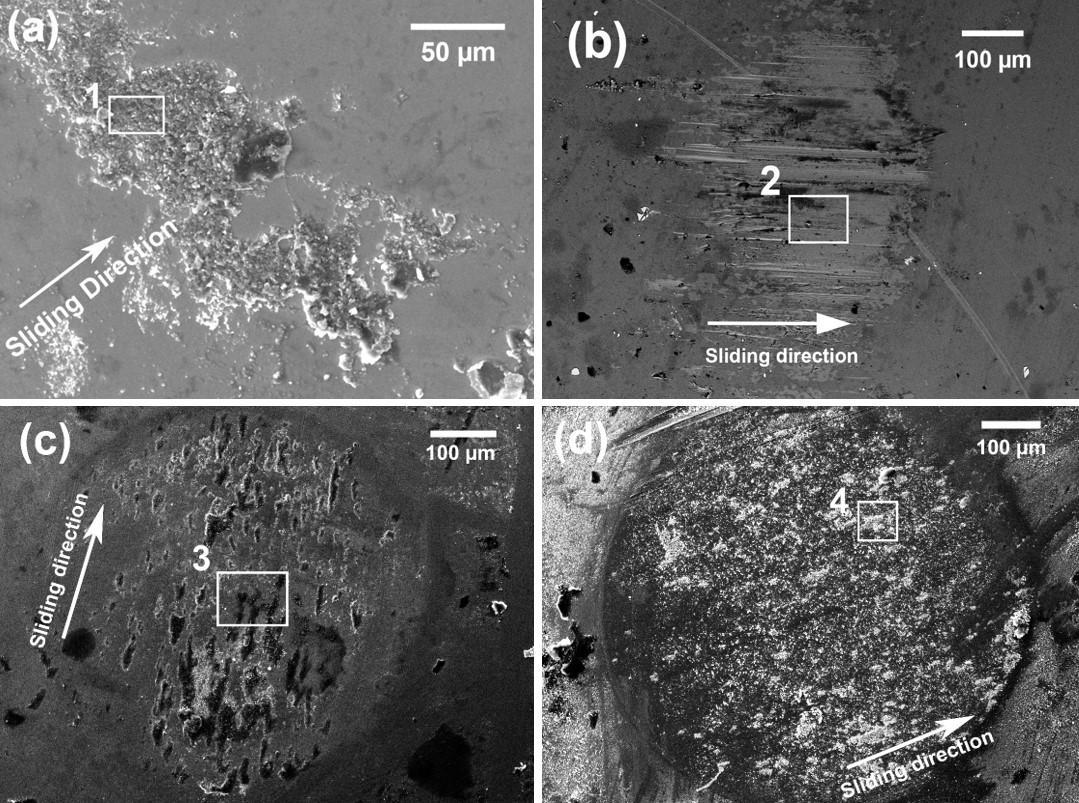** |
| --- |
| Figure S7: EDS sites on wear track of SiOC-ZrSi2 coating at room temperature a) 150 ^o^C; b) 200 ^o^C; c) 300 ^o^C; and d) 400 ^o^C. |

| **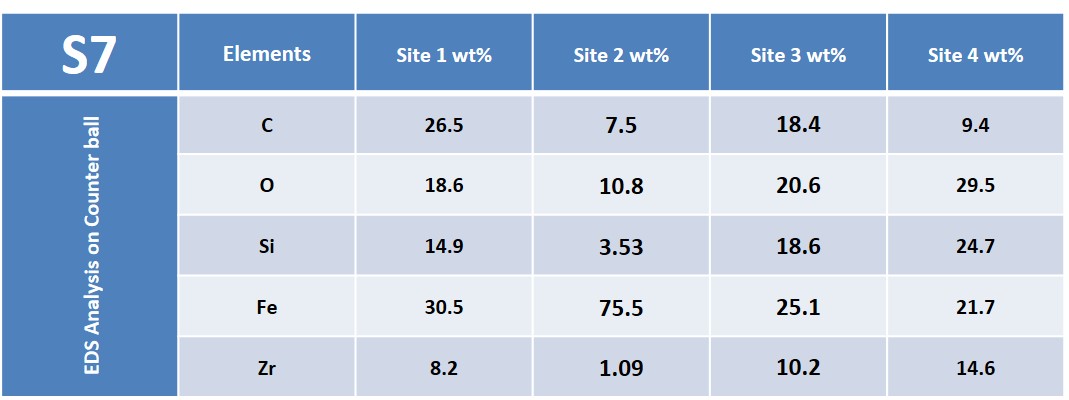** |
| --- |
| Table S7: EDS on steel counter balls from Figure S7 from SiOC-ZrSi_2_ composite coating after 1 N load friction tests at different temperature (site 1) 150 ^o^C; (site 2) 200 ^o^C; (site 3) 300 ^o^C; and (site 4) 400 ^o^C. |

**S8:**

| **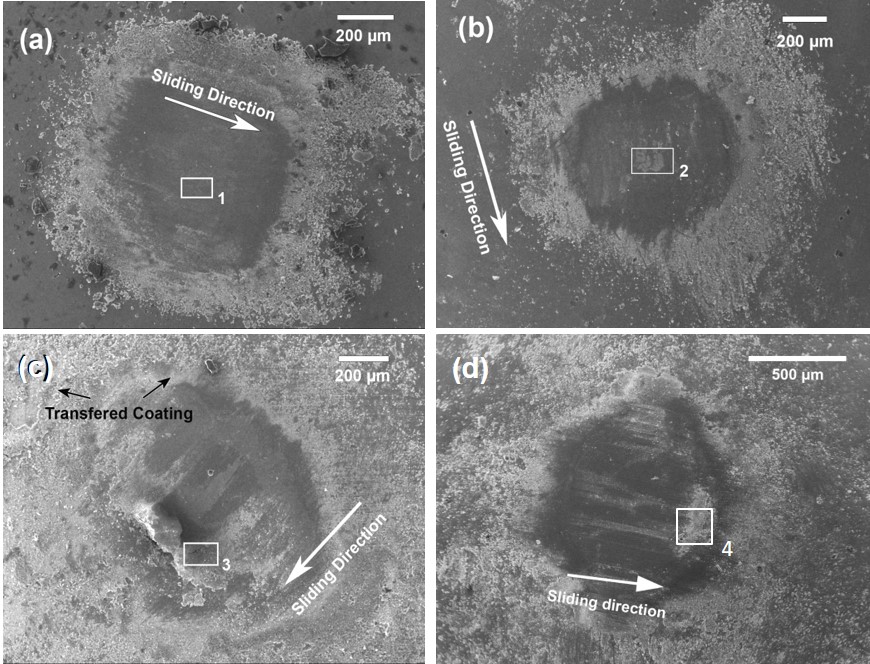** |
| --- |
| Figure S8: SEM images of counter ball from SiOC-Ag composite coating after friction test: (a) 1N load; (b) 2 N load; (c) 3 N load; and (d) 5N. |

| 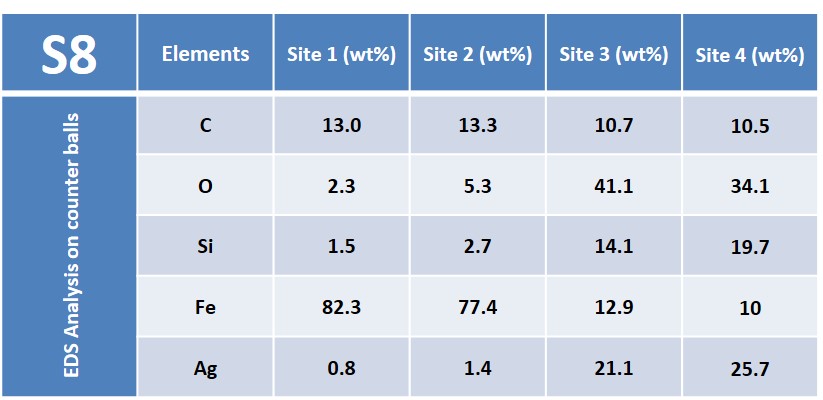 |
| --- |
| Table S8: EDS of counter ball in Figure S8 from SiOC-Ag composite coating after friction test at room temperature: (site 1) 1N load; (site 2) 2 N load; (site 3) 3 N load; and (site 4) 5 N load. |

**S9:**

| 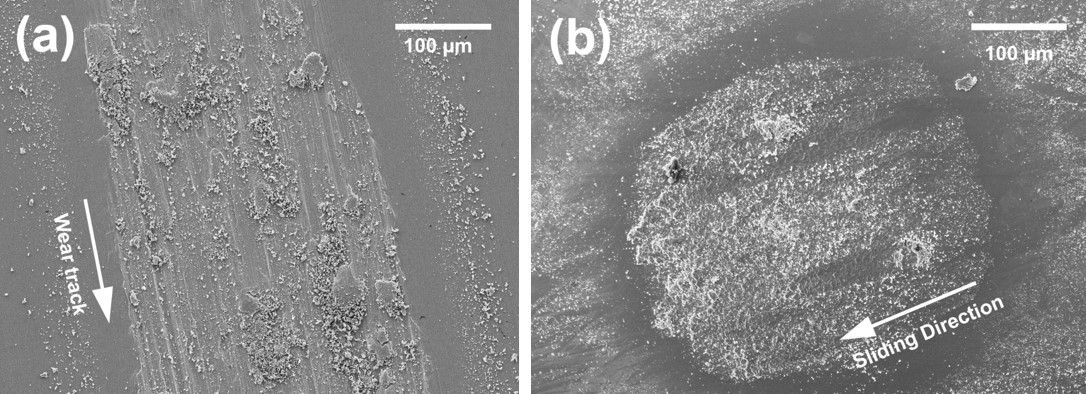 |
| --- |
| Figure S9: SEM images of steel-on-steel friction test at 1N load: (a) wear track; and (b) counter ball |

**S10:**

| 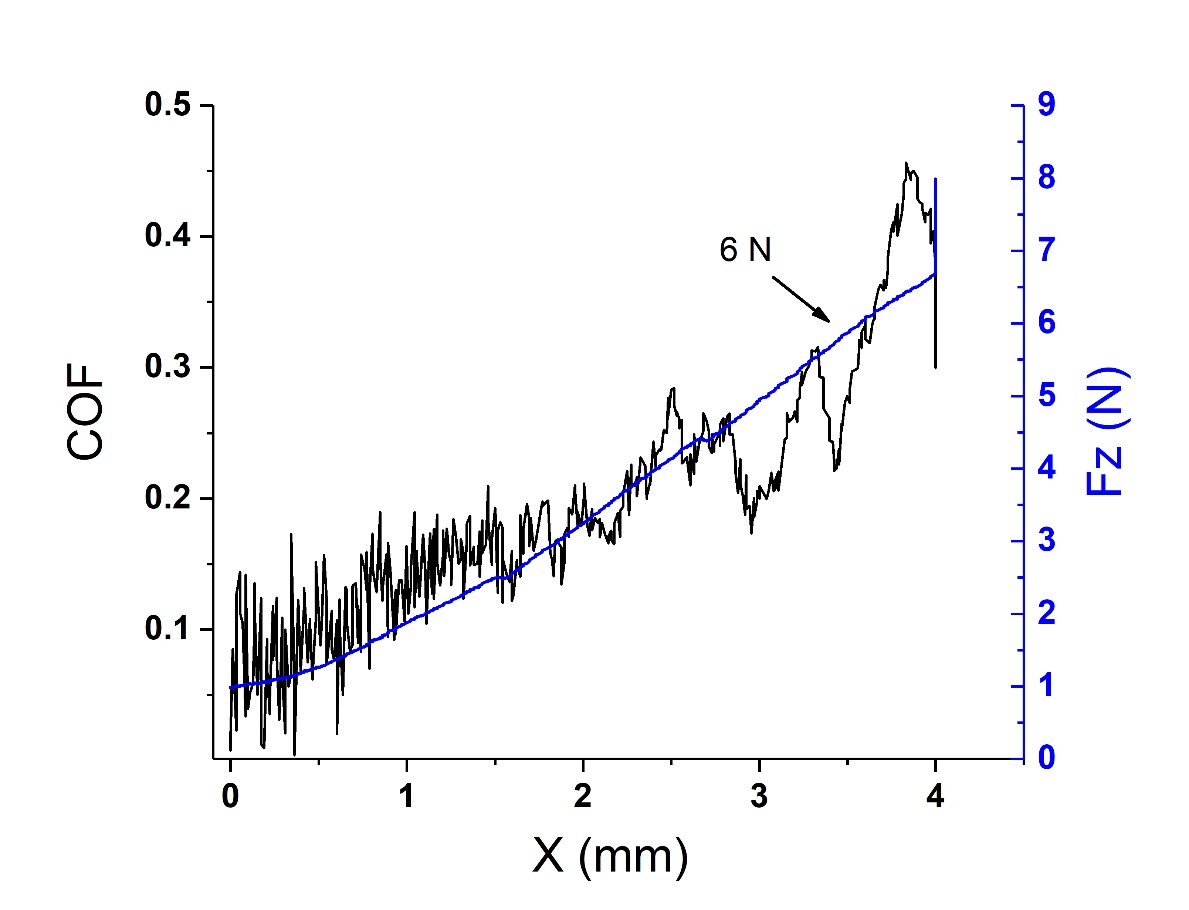 |
| --- |
| Figure S10: Plot of COF and normal load (Fz) versus the sliding distance in x-direction on SiOC-ZrSi_2_ composite coating with increasing normal load from 1 to 8N to observe the adhesion strength.  **S11**   \| 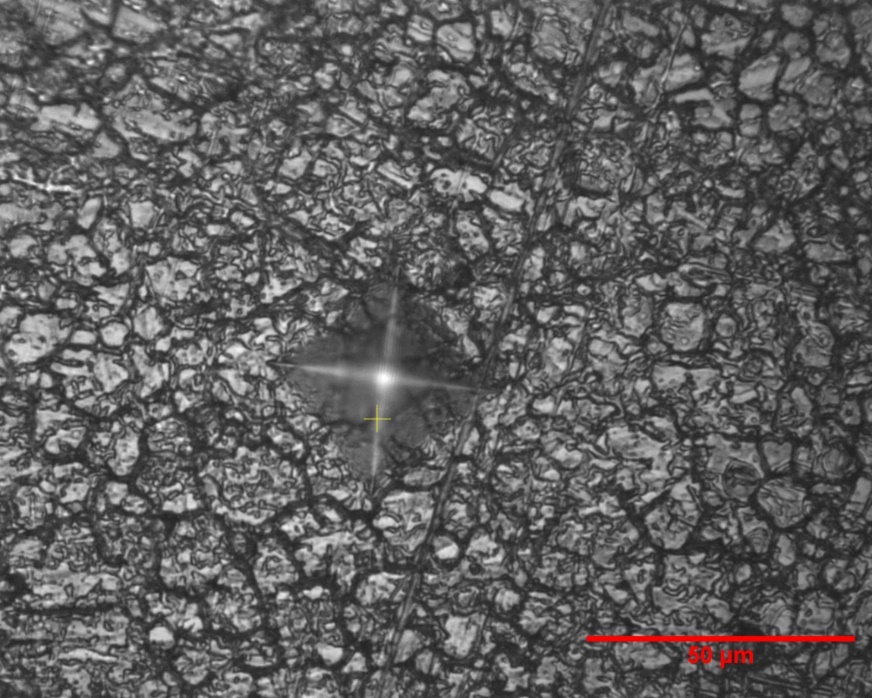 \| \| --- \| \| Figure S11: Vickers microhardness indent on SiOC-ZrSi_2_ composite coating \| |

**S12:**

| **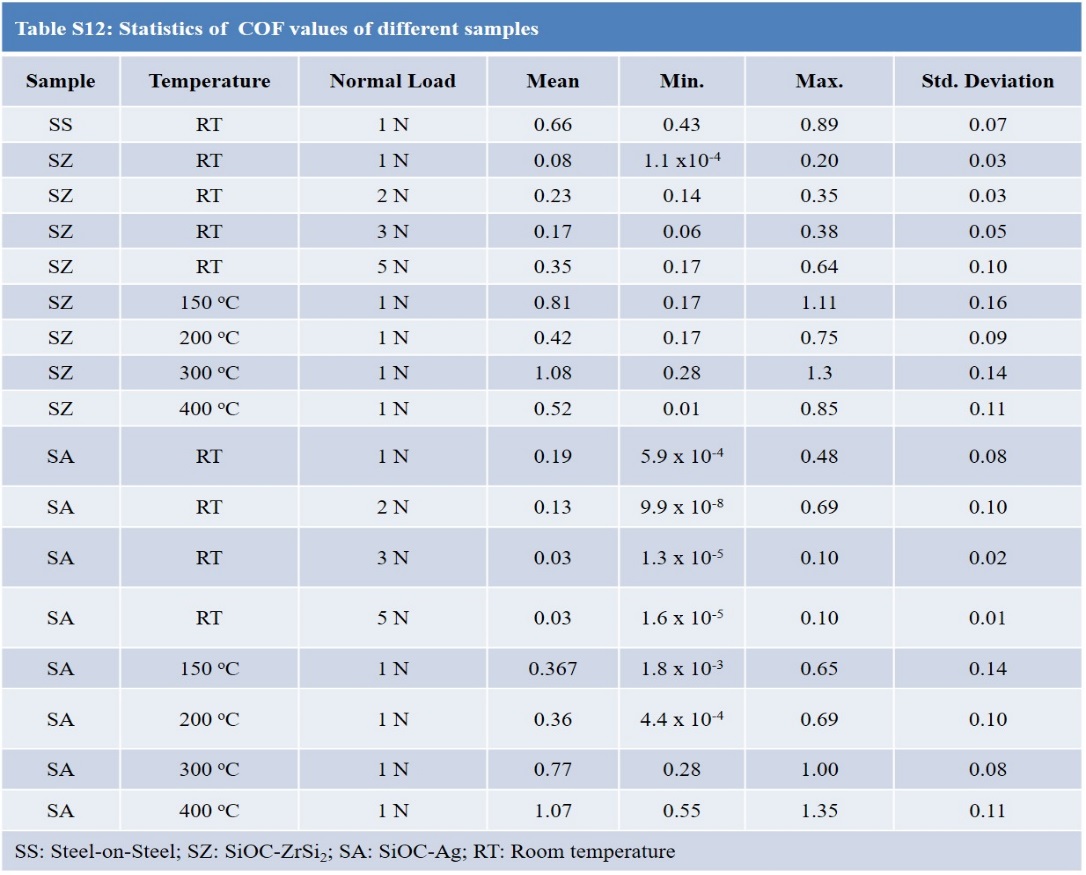** |
| --- |
| Table S12: showing the statistic of COF for different samples. |
